# 16S rRNA/rRNA Gene Ratios and Cell Activity Staining Reveal Consistent Patterns of Microbial Activity in Plant-Associated Soil

**DOI:** 10.1128/mSystems.00003-19

**Published:** 2019-04-02

**Authors:** Alan W. Bowsher, Patrick J. Kearns, Ashley Shade

**Affiliations:** a Department of Microbiology and Molecular Genetics, Michigan State University, East Lansing, Michigan, USA; b Plant Resilience Institute, Michigan State University, East Lansing, Michigan, USA; c Program in Ecology, Evolution, and Behavior, Michigan State University, East Lansing, Michigan, USA; d DOE Great Lakes Bioenergy Research Center, Michigan State University, East Lansing, Michigan, USA; UMR1136 INRA Université de Lorraine

**Keywords:** 16S rRNA, 5-cyano-2,3-ditolyl tetrazolium chloride, beta diversity, community dynamics, dormancy, flow cytometry, microbiome, phantom taxa, resuscitation, rhizosphere

## Abstract

At any given time, only a subset of microbial community members are active in their environment. The others are in a state of dormancy, with strongly reduced metabolic rates. It is of interest to distinguish active and inactive microbial cells and taxa to understand their functional contributions to ecosystem processes and to understand shifts in microbial activity in response to change. Of the methods used to assess microbial activity-dormancy dynamics, 16S rRNA/rRNA gene amplicons (16S ratios) and active cell staining with 5-cyano-2,3-ditolyl tetrazolium chloride (CTC) are two of the most common, yet each method has limitations. Given that *in situ* activity-dormancy dynamics are proxied only by laboratory methods, further study is needed to assess the level of agreement and potential complementarity of these methods. We conducted two experiments investigating microbial activity in plant-associated soils. First, we treated corn field soil with phytohormones to simulate plant soil stress signaling, and second, we used rhizosphere soil from common bean plants exposed to drought or nutrient enrichment. Overall, the 16S ratio and CTC methods exhibited similar patterns of relative activity across treatments when treatment effects were large, and the instances in which they differed could be attributed to changes in community size (e.g., cell death or growth). Therefore, regardless of the method used to assess activity, we recommend quantifying community size to inform ecological interpretation. Our results suggest that the 16S ratio and CTC methods report comparable patterns of activity that can be applied to observe ecological dynamics over time, space, or experimental treatment.

**IMPORTANCE** Although the majority of microorganisms in natural ecosystems are dormant, relatively little is known about the dynamics of the active and dormant microbial pools through both space and time. The limited knowledge of microbial activity-dormancy dynamics is in part due to uncertainty in the methods currently used to quantify active taxa. Here, we directly compared two of the most common methods (16S ratios and active cell staining) for estimating microbial activity in plant-associated soil and found that they were largely in agreement in the overarching patterns. Our results suggest that 16S ratios and active cell staining provide complementary information for measuring and interpreting microbial activity-dormancy dynamics in soils. They also support the idea that 16S rRNA/rRNA gene ratios have comparative value and offer a high-throughput, sequencing-based option for understanding relative changes in microbiome activity, as long as this method is coupled with quantification of community size.

## Abstract (precis)

Although the majority of microorganisms in natural ecosystems are dormant, relatively little is known about the dynamics of the active and dormant microbial pools through both space and time. The limited knowledge of microbial activity-dormancy dynamics is in part due to uncertainty in the methods currently used to quantify active taxa. Here, we directly compared two of the most common methods (16S ratios and active cell staining) for estimating microbial activity in plant-associated soil and found that they were largely in agreement in the overarching patterns. Our results suggest that 16S ratios and active cell staining provide complementary information for measuring and interpreting microbial activity-dormancy dynamics in soils. They also support the idea that 16S rRNA/rRNA gene ratios have comparative value and offer a high-throughput, sequencing-based option for understanding relative changes in microbiome activity, as long as this method is coupled with quantification of community size.

## INTRODUCTION

Microbial activity plays a major role in processes that support life on Earth (1), influencing global carbon and nutrient cycling (2, 3), atmospheric composition (4), and ecosystem productivity (5). Given these global-scale functions, it is perhaps surprising that active microbes (those that are growing or reproducing or that respond relatively quickly to substrate input) represent a small proportion of the total microbial community (reviewed in reference 6). In diverse ecosystems, the majority of the microbial community is dormant, characterized by strongly reduced metabolic rates and a slow response to substrate input (6, 7). Although dormancy initiation and resuscitation have ecological and evolutionary consequences (7–9) with implications for ecosystem function (10, 11), we know little about the dynamics of active and dormant microbial pools through space and time. Investigations of the causes and consequences of microbial activity-dormancy dynamics are needed to better understand community structural and functional resilience and to better predict responses to global change (12).

The limited knowledge of microbial activity-dormancy dynamics is in part due to uncertainty in the methods currently used to quantify active taxa (6). One of the most common approaches is the use of 16S ribosomal rRNA sequencing. Given the relatively short half-life of rRNA, the presence of 16S ribosomal transcripts (hereinafter “rRNA”) is generally assumed to indicate recent metabolic activity, and numerous studies have used rRNA to characterize active communities (reviewed in reference 13). Pairing both 16S rRNA and 16S rRNA gene sequencing allows for calculation of 16S rRNA/rRNA gene ratios (herein “16S ratios”), which attempts to normalize rRNA levels by the abundances of individual taxa in the community and quantify their relative levels of activity (9, 11, 14, 15). Taxa with 16S ratios greater than a given threshold are considered active; most studies use a threshold of 1.0. (6, 9, 16). However, using an arbitrary threshold to distinguish active from dormant taxa may be problematic in diverse communities (13, 17), given that rRNA content or RNA/DNA ratios and growth rate are not always correlated (18–22). Another challenge is the occurrence of “phantom taxa,” taxa that are detected in rRNA but not rRNA gene sequences (23), leading to a zero denominator (and thus undefined) 16S ratio. Phantom taxa are unexpected, since the presence of rRNA necessitates a template rRNA gene, yet nearly 30% of the operational taxonomic units (OTUs) detected in a recent study of atmospheric samples were phantoms (23). Although these considerations have led researchers to suggest that 16S ratios may be best interpreted as potential microbial activity, 16S ratios have nevertheless been used to inform microbial activity-dormancy dynamics in a broad range of ecosystems (11, 14, 15, 23).

In addition to 16S rRNA/rRNA gene sequencing, a variety of methods are currently used for distinguishing active microbes. These include staining with tetrazolium salts (reference 24 and references therein), stable-isotope probing to quantify uptake of substrates or water (25), and meta-transcriptomics to determine changes in functional gene transcripts following experimental perturbation (26). Of these methods, active-cell staining, primarily with the activity stain 5-cyano-2,3-ditolyl tetrazolium chloride (CTC), is a common approach because it is economical and executable without specialized equipment. Respiring cells convert CTC to an insoluble red fluorescent formazan product that can be visualized by fluorescence microscopy (27). In addition, CTC staining can be coupled with a DNA stain to compare active and total cell counts in a microbial community, allowing for determination of percentages of activity (15, 28). Numerous culture-dependent studies have shown that the CTC method is effective in discriminating between active and inactive microbes (29–32) and between growth phases (29, 30) and can track resuscitation following starvation (32). The CTC method also can reveal subtle changes in activity while quantifying changes in the total number of cells (active plus inactive) to provide context for interpreting the activity dynamics. For example, in Pseudomonas fluorescens (32), only ∼10% of cells were active after starvation in phosphate-buffered saline. However, the number of CTC-positive cells increased after a 5-h incubation with yeast extract, without a corresponding increase in total cell number (32). This apparent resuscitation of cells from CTC negativity to CTC positivity after nutrient addition showed that CTC staining is capable of discriminating between active cells (high metabolic activity) and dormant cells (low metabolic activity) even when doubling is not observed. Nevertheless, CTC can be toxic to some bacterial species (24, 33), and not all actively respiring strains are able to take up the stain efficiently (24, 28), potentially leading CTC staining to underestimate the true proportion of active cells (34). Variability in the staining protocol (e.g., staining durations and concentrations of stain) also can have consequences for comparing percentages of activity across different studies (29). Despite these limitations, CTC staining remains a popular method for analyses of microbial activity in a broad range of environmental samples (7, 15, 28).

Although both the 16S ratio method and the CTC staining method are commonly used in investigations of microbial activity, little work has been done to assess their level of agreement. One of the few studies using both methods to assess microbial activity found that the active portion of the community was between 1.5- and 5-fold higher when 16S ratios were used than when CTC staining was used in microcosms of estuarine water samples (15). One potential reason is not only that the two methods present different biases as described above but that they measure two different things: the 16S ratio method is used to assess whether a particular taxon is active, while the CTC staining method is used to assess whether a given cell is active. Importantly, there are situations in which we might expect the proportion of active taxa and the proportion of active cells to differ, such as in communities in which rare taxa are disproportionately active compared to abundant taxa (15, 23, 35). Another potential discrepancy between the 16S ratio and the CTC staining method is their respective definitions of “active” and “inactive,” since RNA and DNA levels do not always correlate with respiration rates (36). Although these two methods may not always produce similar values of microbial activity, both can inform on fundamental and complementary aspects of microbial communities.

Here, our objectives were to explore factors underlying the calculation and interpretation of 16S ratios and to compare estimates of the activity of microbial communities using 16S ratios and CTC-based cell staining. We conducted two experiments analyzing microbial activity in plant-associated soil. Given that plant-associated soils are highly dynamic systems in which plants can influence microbial community structure and function (37, 38), we considered plant-associated soil to be particularly relevant for analyses of microbial activity. First, we conducted a microcosm experiment using corn-associated soil and treated the soil with phytohormones to assess the impacts of common plant stress signals on soil microbial activity. Second, we grew bean plants under either drought or nutrient-enriched conditions to more directly assess the impacts of plant stress on soil microbial activity. Specifically, we asked the following. (i) For 16S ratio-based studies, what is the extent of phantom taxa, and how does the handling of these taxa influence estimates of microbial activity and patterns across treatments? (ii) How does the threshold for quantifying active taxa influence patterns across treatments? (iii) How does 16S rRNA operon copy number impact the distribution of 16S ratios within and across phyla? (iv) Do 16S ratio and CTC methods produce similar estimates and/or patterns of microbial activity across diverse soil treatments?

## RESULTS AND DISCUSSION

We conducted two experiments with plant-associated soils under a variety of treatment conditions. In the first experiment, we collected soil from a long-term agricultural research field in which corn (*Zea mays* L.) had been planted for eight consecutive years. In the laboratory, we exposed the soil to a few treatments before it was sampled: the pre-dry treatment (soil was sampled before any treatments were initiated), post-dry treatment (soil was dried for 3 days and then sampled), and post-water treatment (soil was partially rewetted, allowed to acclimate for 6 days, and then sampled). Next, soil replicates were treated with either abscisic acid (ABA), indole-3-acetic acid (IAA), jasmonic acid (JA), salicylic acid (SA), or water as a control and sampled after 24 h. Thus, the corn experiment was designed to assess the impacts of several different abiotic/biotic stresses, including soil drying and rewetting, as well as the application of common plant stress phytohormones that can be exuded by plant roots under a variety of stress conditions (39). In the second experiment, we grew common bean (Phaseolus vulgaris L. cv. Red Hawk) in agricultural field soil in a controlled-environment growth chamber. Replicate plants were exposed to either drought (water withholding), nutrient enrichment (additional fertilizer), or control conditions, and then rhizospheres were sampled after 5 weeks. Thus, this experiment was designed to more directly assess the impacts of plant stress on analyses of soil microbial activity in plant-associated soil. Overall, we anticipated that the differential treatments within and across experiments, as well as the presence of actively growing plants continuously providing carbon to the soil microbial communities in the bean but not the corn soil study, would inform on the broad applicability of the 16S rRNA and CTC staining methods for comparing microbial activities.

### Detection and treatment of phantom taxa.

A necessary prerequisite for assessing microbial activity from 16S ratios is determining how to handle phantom taxa, OTUs that are detected in the RNA but not the DNA community of a given sample (23). Phantom taxa can occur for both biological and methodological reasons, such as the sampling stochasticity of the “rare biosphere” when rare taxa exhibit disproportionately high activity (23). In addition, different methods of RNA and DNA extraction or biases, such as the reverse transcription of RNA but not of DNA, might contribute (40). Finally, heterogeneity between the soil aliquots used for RNA and those used for DNA extraction might lead to different community profiles in the two extractions and therefore lead to phantom taxa (in the present study, soil was mixed prior to DNA/RNA extractions to reduce this bias). Regardless of the mechanism of their occurrence, phantom taxa cannot be avoided when using 16S ratios.

Phantom taxa are problematic because they produce undefined 16S ratios due to a denominator of zero and, without care, could be automatically deleted from the data set. Therefore, we assessed the prevalence of phantom taxa (taxa with RNA reads of >0 and DNA reads equal to 0 in a given sample) in both the corn and the bean soil data sets. We also assessed the prevalence of singleton phantom taxa (taxa with RNA reads equal to 1 and DNA reads equal to 0 in a given sample), given that such taxa are particularly ambiguous in terms of activity. We repeated these analyses across a range of sequencing depths, given that sampling stochasticity can play a role in driving the occurrence of phantom taxa (23). Across a range of subsampling levels, we found that phantom taxa comprised 6 to 62% of the total OTUs in the corn soil data set and 17 to 41% of the total OTUs in the bean soil data set (see Fig. S1A and B and S2A and B in the supplemental material). Similarly, singleton phantom taxa were fairly common (1 to 38% and 4 to 27% of the total OTUs in the corn and bean soil data sets, respectively) (Fig. S1C and D and S2C and D). The reader should note that the sample size for each treatment generally decreased as sequencing depth increased because samples were excluded when their total read count was less than a given sequencing depth.

10.1128/mSystems.00003-19.1 FIG S1 Prevalence of taxa with 16S RNA reads but zero 16S DNA reads (A, B) (i.e., phantom taxa) or a single 16S RNA read and zero 16S DNA reads (C, D) in soil associated with corn (A, C) and bean (B, D) as a function of sequencing subsampling level. Shown are best-fit lines using the LOESS smoothing function (see Fig. S2 for the same plots but including individual data points). Gray shading around the smoothing lines are 95% confidence intervals. Download FIG S1, PDF file, 0.3 MB. Copyright © 2019 Bowsher et al. 2019 Bowsher et al. This content is distributed under the terms of the Creative Commons Attribution 4.0 International license.

10.1128/mSystems.00003-19.2 FIG S2 Prevalence of taxa with 16S RNA reads but zero 16S DNA reads (A, B) (i.e., phantom taxa) or a single 16S RNA read and zero 16S DNA reads (C, D) in soil associated with corn (A, C) and bean (B, D) as a function of sequencing subsampling level. Points indicate individual samples, with best-fit lines using the LOESS smoothing function. Gray shading around the smoothing lines are 95% confidence intervals. See Fig. S1 for the same plots but including only smoothing lines and confidence intervals. Download FIG S2, PDF file, 0.3 MB. Copyright © 2019 Bowsher et al. 2019 Bowsher et al. This content is distributed under the terms of the Creative Commons Attribution 4.0 International license.

**FIG 1:**
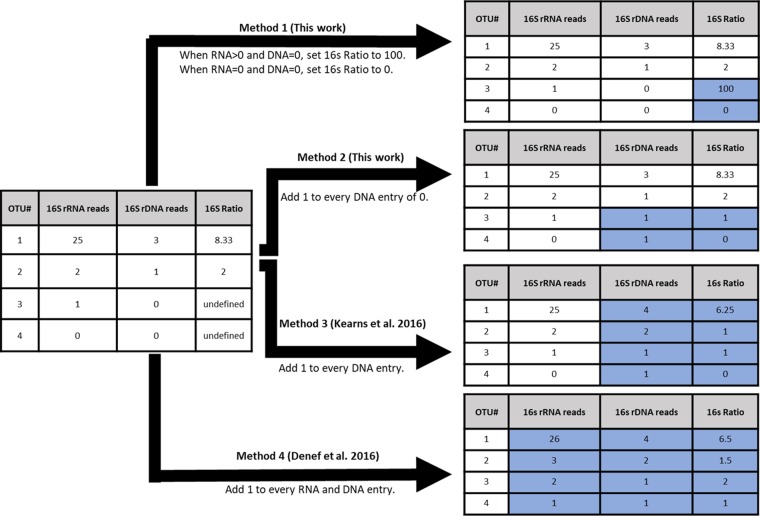
Conceptual diagram depicting the impacts of four distinct methods for calculating 16S rRNA/rRNA gene ratios in the presence of phantom taxa (i.e., OTUs in a given sample with 16S RNA reads but zero 16S DNA reads, producing an undefined 16S ratio due to a zero denominator). The input OTU table for a given sample along with 16S ratios is shown on the left, while the resulting OTU tables and 16S ratios are depicted on the right (changes are shaded blue). Four different sequencing scenarios in a hypothetical sample are considered: OTU1, in which the number of RNA reads is much larger than the number of DNA reads but both are present; OTU2, in which the number of RNA and DNA reads are both low but present; OTU3, in which the number of RNA reads is one and the number of DNA reads is zero; and OTU4, in which the number of both RNA and DNA reads is zero.

**FIG 2:**
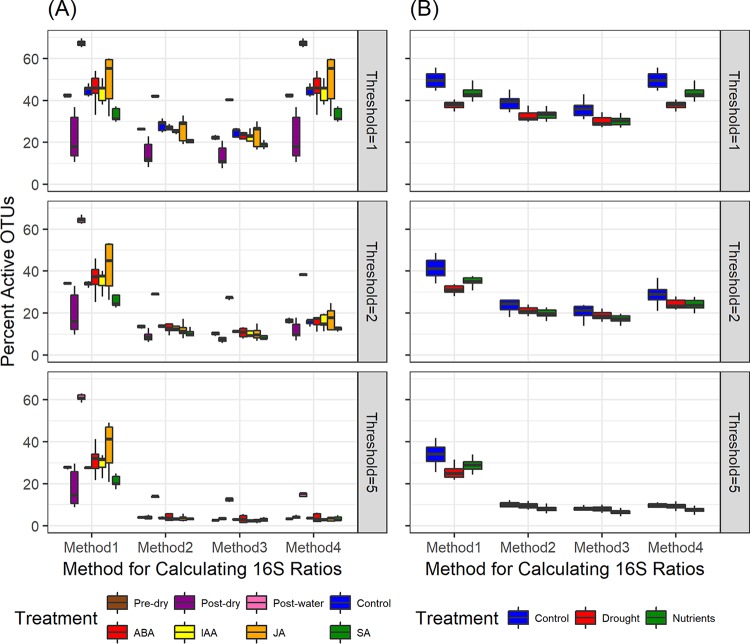
Comparison of the proportions of taxa that are active (i.e., the percentage of total OTUs with a 16S rRNA/rRNA gene ratio greater than a given threshold) in soil associated with corn (A) and bean (B) following each of four methods for calculating 16S ratios. See Fig. 1 for a depiction of the four methods for calculating 16S ratios and the text for a description of treatment conditions.

Given the prevalence of phantom taxa, our next aim was to establish how to handle phantom taxa for calculation of 16S ratios. We compared four different methods for calculating 16S ratios in the presence of phantom taxa, referred to here as methods 1, 2, 3, and 4 for simplicity (Fig. 1). In both the corn and the bean soil data sets, all four methods for calculating 16S ratios produced similar patterns across treatments (Fig. 2A and B). In corn soil, percent activity decreased from the pre-dry to the post-dry treatment and then increased from the post-dry to the post-water treatment (Fig. 2A). In addition, although increasing the threshold 16S ratio for defining taxa as active generally reduced the magnitude of treatment effects on microbial activity, the large impact of the post-water treatment was apparent even at a threshold of 5. Similar analyses have been performed in in other studies (11), and they suggest that even conservative ratio thresholds provide access to consistent ecological patterns. On the other hand, although percent activity in the bean experiment was generally higher in the control than in the drought or nutrient treatments across methods 1 to 4, the magnitude of this difference decreased as threshold increased (Fig. 2B). Differences among treatments disappeared when they were compared at a threshold 16S ratio of 5, indicating a relatively narrow window for capturing differences in microbial activity in the bean experiment. A recent review suggests that most studies use a threshold 16S ratio between 0.5 and 2 to determine active taxa (6), indicating that a threshold of 5 might simply provide an overly conservative view of activity in microbial communities.

### Weak relationship between the 16S ratio and the number of ribosomal operons in genomes.

**FIG 3:**
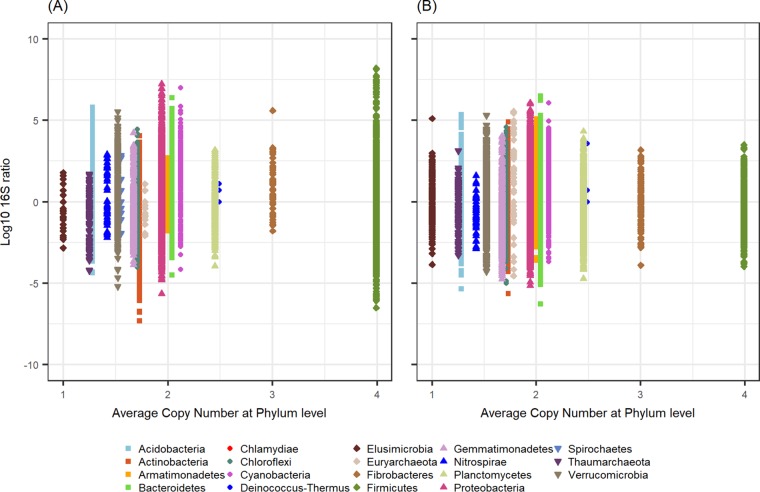
16S rRNA/rRNA gene ratio as a function of the average 16S operon copy number for the presented phyla detected in corn rhizosphere (A) and bean-associated soil (B), as determined by the Ribosomal RNA Database (*rrn*DB). Data points represent every occurrence (i.e., within and across all samples) for a given phylum. Only phyla with representatives in the *rrn*DB are shown. Note that the phylum *Spirochaetes* was present only in corn-associated soil.

One consideration of using 16S ratios to estimate the proportion of the active-taxon community is the variability in copy numbers of the 16S rRNA operon across genomes of different taxa. 16S rRNA operon copy numbers can affect patterns of beta diversity in community structure (41) and can vary substantially between lineages (42). For example, lineages with many 16S operons (e.g., firmicutes) might have lower 16S ratios because their abundance is overestimated by redundant reads in 16S rRNA gene data. To address this, we examined the relationship between the 16S ratio and the average number of ribosomal operons within phyla for all detected OTUs (Fig. 3). Although 16S ratios and average 16S rRNA operon count at the phylum level were correlated in both corn (*r* = −0.069, *P < *0.0001) (Fig. 3A) and bean (*r* = −0.017, *P* = 0.0001) (Fig. 3B) soil, these correlations were weak, to the point of being inconsequential for the interpretation of overarching patterns at the community level. In addition, across all operon counts, 16S ratios had similar ranges (Fig. 3). Recent work has advised against correcting for 16S rRNA operon counts in 16S rRNA gene surveys of microbial community structure, especially in taxa that are divergent from cultured representatives (41). Our results additionally suggest that accounting for 16S operon number will likely have little effect on activity estimates in 16S ratio analyses.

### CTC staining and 16S ratios capture complementary patterns of activity across treatments.

**FIG 4:**
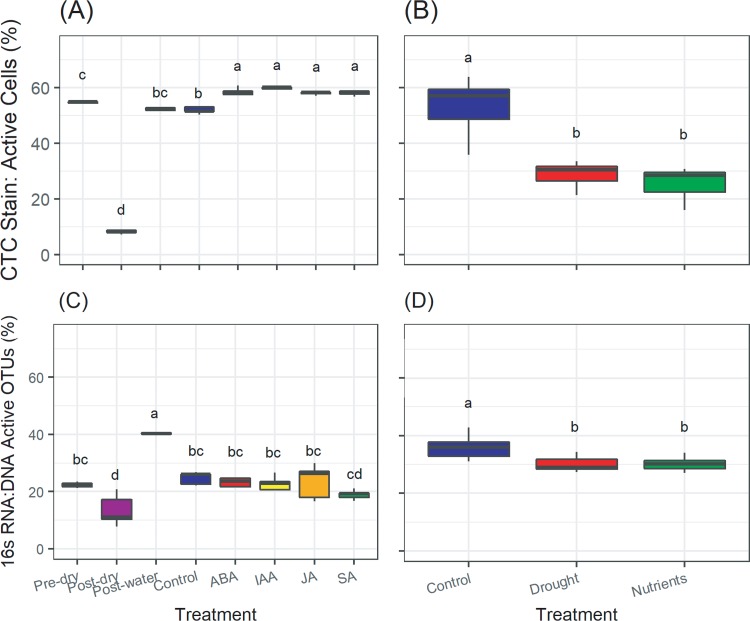
Proportions of active taxa/cells as determined by CTC/Syto24 staining (A, B) or 16S rRNA/rRNA gene ratios (C, D) in soil associated with corn (A, C) and bean (B, D). Taxa in panels C and D are defined as active, with a 16S rRNA/rRNA gene ratio of >1. See the text for a description of treatment conditions.

Our final aim was to assess the level of agreement between the 16S ratio and the CTC method (Fig. 4). Across all treatments and between both methods, estimates of percent activity (i.e., between 10 and 60% of cells/taxon are active) are similar to values reported in the literature for soil (7). Though, as expected for the reasons discussed in the introduction, the two methods did not agree in their absolute values of percent activity, their overarching patterns across treatments were largely consistent, suggesting that either method is appropriate for assessing overarching patterns in community activity (e.g., across time, space, or experimental treatments). The CTC method consistently resulted in higher estimates of activity than the 16S ratio method.

In bean rhizosphere soil, the CTC method and the 16S ratio method produced identical patterns of percent activity across treatments (Fig. 4B and D). Using both methods, the drought and the nutrient addition treatments exhibited lower percentages of activity than the control treatment, although the magnitude of this difference was less using the 16S ratio method than the CTC method. In corn-associated soil, both the CTC staining method and the 16S ratio method revealed similar patterns in response to corn soil drying and wetting: percent activity declined from the pre-dry to the post-dry treatment and increased in response to the post-water treatment. Together with the bean rhizosphere experiment, these results suggest that both methods are consistent in detecting relative activity changes when there are large treatment effects.

In the corn soil, there were method differences in activity from the post-water and control treatments (Fig. 4A and C). Using the CTC method, percent activity increased in response to phytohormones compared to that of the post-water treatment but did not change in response to the control treatment (i.e., water alone) (Fig. 4A). Using the 16S ratio method, percent activity decreased in response to phytohormones as well as in the water control compared to that of the post-water treatment (Fig. 4C). For these subtle activity changes observed post-water treatment in the corn-associated soil, the differences between the 16S ratio and CTC methods could be explained by the shifts in community sizes across treatments. Though CTC percent activity did not change post-water treatment relative to that of the control treatment, raw cell counts measured by Syto24 aid in interpretation. Both CTC counts (i.e., the number of active cells) and Syto24 counts (i.e., the total number of cells regardless of activity) decreased to similar extents from the post-water to the control treatment (20.3% decrease versus 19.7% decrease, respectively) (Fig. S3A and C), resulting in no change in percent activity despite the decrease in community size. The drop in the proportion of active OTUs by the 16S ratio but not the proportion of cells by CTC from post-water to control is likely due to cell death and resultant changes in community size that are not accounted for when using 16S ratios (and might be exacerbated by the contributions of DNA from recently dead cells to the denominator). Similarly, CTC counts were slightly lower in the phytohormone treatments than in the control (average difference of 2.9%), but Syto24 counts were much lower in the phytohormone treatments than in the control (average difference of 14.0%) (Fig. S3A and C), which explains the increased percent CTC activity in phytohormone treatments versus control treatments. However, despite the decrease in community size (i.e., cell death) between phytohormone and control treatments (Fig. S3C), percent activities using the 16S ratio method did not differ between phytohormone and control samples, potentially highlighting the different metrics reported by the CTC method (percentage of cells that are active) and the 16S ratio method (percentage of taxa that are active). Overall, there is immense value added by measuring changes in community size when interpreting changes in activity, regardless of the method applied. Future studies using the 16S ratio method for assessing activity dynamics should consider using cell counting or an equivalent method for approximating community size (e.g., quantitative PCR) coupled with propidium monoazide treatment to block amplification of DNA from dead/dying cells (e.g., see reference 43).

10.1128/mSystems.00003-19.3 FIG S3 Flow cytometer counts (i.e., the number of cells counted) per gram of soil extracted following staining with CTC (A, B) and Syto24 (C, D). Download FIG S3, PDF file, 0.1 MB. Copyright © 2019 Bowsher et al. 2019 Bowsher et al. This content is distributed under the terms of the Creative Commons Attribution 4.0 International license.

Intense disturbances can result in cell death, and for these events, it is expected that changes in percent activity might mirror changes in microbial community size (total number of cells). Examples of such disturbances include those in the present study (desiccation and phytohormone exposure), antibiotic treatment, oxygen depletion, exposure to predators or phage, and irradiation. There are also disturbances that may be expected to stress cells and alter phenotypes but not to cull a significant proportion of the community. Therefore, the ecological context is of paramount importance for interpreting activity dynamics, and measurements of community size can inform as to the outcomes of disturbance in situations that have unclear expectations *a priori*.

### Considerations of the 16S ratio and CTC staining methods.

An important consideration of this work is that both the 16S ratio and CTC methods have distinct biases that can influence percent activity estimates. A number of issues have been highlighted for analysis of activity with 16S ratios (13, 17, 44, 45), including the presence of dead cells and extracellular DNA, variations in sequencing depth, and molecular methodology (PCR biases). Another issue is the inconsistent relationships between rRNA and growth rate described above and the finding that cellular rRNA can actually increase in the transition from the vegetative to the dormant state for some taxa (46). Although CTC staining avoids many of these assumptions, it has its own unique biases. For example, CTC staining in the present study excluded obligate anaerobes, potentially underestimating percent activity. In addition, CTC staining assumes that all (or a representative subsample) of the cells are extracted from the soil, an assumption that may or may not be true (47). Finally, because extraction and CTC staining can last for up to 24 h, artifacts such as cell death or changes in respiration rates might occur. Numerous methods, each with a unique set of advantages and limitations (reviewed in reference 6), exist to estimate microbial activity in soil.

Another consideration of our analysis is the inability of the methods used here to account for extracellular DNA and dead cells in soil. Extracellular DNA (48, 49) and necromass (50) are common, and can cause 16S ratios to underestimate percent activity (44). Similarly, although our flow cytometry size gating likely excludes extracellular DNA by removing particles of <1 µm, intact dead cells would be included in the total cell count calculated by Syto24 staining, thereby underestimating percent activity. Although our study was not designed to allow determination of extracellular DNA or dead cell abundance, we note that plant-associated soils are generally assumed to be areas of high metabolic activity. Thus, we might expect relic nucleic acids or dead cells to turn over quickly, limiting their confounding effects in the present study. Our data support the rapid turnover of dead cells, given that the control (water) and phytohormone treatments in the corn soil experiment, which lasted only 24 h, led to significant decreases in total cell counts (Fig. S3C). It should also be noted that, in the corn soil experiment, soils were frozen prior to activity analyses, potentially increasing the number of dead cells and artificially reducing percent activity. We suggest that this impact was minimal, given that percent activity was high compared to previous estimates in soil (7). Nevertheless, we suggest that combining the 16S ratio and CTC/Syto24 approach with a stain specific to extracellular DNA and dead/dying cells, such as propidium iodide, would clarify the impact of these pools (51).

As described above, the dynamic range of the CTC method is expected to be high: over 90% of cells have been reported as CTC positive in pure cultures in exponential growth phase (31), while less than 10% are CTC positive when dormancy is induced by overnight starvation conditions (32). Our findings suggest that the CTC staining method is potentially more sensitive than the 16S ratio method and may be more appropriate for detecting subtle activity changes. For example, the CTC staining method detected larger shifts in percent activity than the 16S ratio method in response to the drying (46.1% decrease versus 8.8% decrease, respectively) and rewetting (43.8% increase versus 27.3% increase, respectively) treatments of corn-associated soil (Fig. 4A and C) and in response to the drought (23.1% decrease versus 5.8% decrease, respectively) and nutrient addition (27.6% decrease versus 5.5% decrease, respectively) treatments of bean soil (Fig. 4B and D). Similarly, the CTC method detected significantly higher percent activity in all phytohormone treatments than in the control, while the 16S ratio method did not detect a difference between the control and phytohormone treatments (Fig. 4A and C). On the other hand, the 16S ratio method detected a much larger difference from the post-water to the control (i.e., water added) treatment (16.0% decrease versus 0.3% decrease, respectively) (Fig. 4A and C). Altogether, these shifts in percent activity across treatments are in the range of those reported in the literature. For example, a study using the CTC staining method to assess microbial communities on sandstone found that percent activity values dropped from 60% to 20% after 2 days of drying at low relative humidity (30), similar to the shift in CTC staining in response to soil drying in the present study (Fig. 4A). However, further study is needed to assess the lower limits of detection of the two methods for diverse taxa and inform on their ability to capture more-subtle changes in percent activity.

### Conclusions.

Overall, our results provide insight into the estimation of microbial activity using two common methods: 16S ratios and CTC staining. Although phantom taxa were common, patterns in percent activity across treatments were largely unaffected by the method used to account for such taxa. We also found that 16S ratios were only weakly correlated with ribosomal operon number, suggesting that accounting for operon number has little effect on activity estimates in 16S ratio analyses. Lastly, both the 16S ratio and CTC methods exhibited similar patterns of percent activity across treatments, and the instances in which they differed can be explained largely by changes in community size. We conclude that quantification of community size is essential for interpreting activity dynamics. Coupled with quantification of community size, the two methods provide comparable assessments of relative changes in microbial activity.

## MATERIALS AND METHODS

We conducted two separate experiments (corn-associated soil and bean rhizosphere soil), with various stress treatments in each experiment. For clarity, we first present the methods specific to each experiment, and then present the methods shared between experiments.

### Corn-associated soil: experimental design, sample collection, and preparation for sequencing.

In the first experiment, topsoil was collected on 21 August 2017 from the AGR-Corn treatment of the Great Lakes Bioenergy Resource Center Scale-Up Experiment located near the Kellogg Biological Station, Hickory Corners, MI. Corn has been planted annually at that site since 2010. Replicate soil cores (to a depth of 10 cm) were collected <30 cm from the stalk of corn plants (corn roots were present in the cores) using a 2.5-cm-diameter steel corer, transported to the laboratory on ice, sieved, and homogenized. Three replicate soil aliquots were weighed, dried for 72 h at 70°C, and then reweighed to determine soil percent moisture (mean, 8.6% ± 0.3% [standard deviation]), and the remaining soil was stored at 4°C until use. Soil composition was 56.1% sand, 26.9% silt, and 17% clay as assessed by standard protocols of the Michigan State University Soil and Plant Nutrient Laboratory.

Broadly, the experimental design consisted of several treatments, the pre-dry treatment (soil was sampled before any treatments were initiated), post-dry treatment (soil was dried and then sampled), and post-water treatment (soil was partially rewetted and then sampled), before soils were exposed to one of five treatments, application of the phytohormones abscisic acid (ABA), indole-3-acetic acid (IAA), jasmonic acid (JA), salicylic acid (SA), or water (control). On 2 April 2018, five replicates (30 g each) of the sieved and homogenized soil was retrieved from 4°C storage and frozen at −80°C for DNA/RNA extractions (i.e., the pre-dry treatment). The remaining soil was dried for 72 h at 45°C, at which point another five replicates (30 g each) were stored at −80°C (i.e., the post-dry treatment). The remaining dried soil was split into 50-ml conical tubes (30 g of dry soil each), and each tube received water to achieve 4.3% soil moisture (half of the initial 8.6% soil moisture). This initial wetting step was included to isolate potential responses to phytohormones from the known response to moisture (see reference 10 and references therein). Tubes were vigorously mixed and any clumps broken up with a sterile pipet. Tubes were incubated at room temperature for 6 days, and then five replicates were frozen at −80°C (i.e., the post-water treatment). The remaining tubes were then randomly assigned to one of five treatments: IAA, JA, ABA, SA, or water. Five replicate tubes received 1.12 ml of the appropriate 0.22-µm-filter-sterilized phytohormone dissolved in water at a concentration of 1 mM, while the control tubes received filter-sterilized water alone. Thus, these treatments restored all tubes to the initial 8.6% soil moisture. Tubes were vigorously mixed and clumps broken up with a sterile pipet. After 24 h, the soil samples were frozen at −80°C.

DNA was extracted from ∼0.23-g soil samples using the Qiagen PowerSoil kit by following the manufacturer’s recommendations, while RNA was extracted from a protocol modified from references 11 and 52. Briefly, up to 0.5 g of soil was added to 200 µl of autoclaved PBL buffer (5 mM Na_2_-EDTA, 0.1% [wt/vol] sodium dodecyl sulfate, and 5 mM Tris-HCl; pH ∼ 3) and vortexed for 1 min, and then 1 ml of phenol-chloroform-isoamyl alcohol (25:24:1 [vol/vol/vol], pH 8) was added. Samples were vortexed for 15 min and then centrifuged for 5 min at 20,000 × *g*. The upper (i.e., aqueous) layer was collected, added to 1 ml isopropanol, and vortexed. Samples were centrifuged for 15 min at 20,000 × *g*, and the supernatant was carefully removed. Tubes were air dried for 15 min and then resuspended in 50 µl of sterile water. Resuspended RNA extracts were cleaned using the OneStep PCR inhibitor removal kit (Zymo Research, Irvine, CA).

### Bean rhizosphere soil: experimental design, sample collection, and preparation for sequencing.

In the second experiment, we planted 24 one-gallon pots with the common bean, Phaseolus vulgaris L. (var. Red Hawk), in local Michigan field soil in a controlled-environment growth chamber (BioChambers FXC-19). Soil composition was 73.7% sand, 14.9% silt, and 11.4% clay. Plants received a photoperiod of16 h of light and 8 h of dark, with a daytime temperature of 29°C and a nighttime temperature of 20°C. Eight replicate plants received ample water throughout the course of the experiment and served as controls. Eight additional replicates received ample water with the addition of nutrients (half-strength Hoagland solution [53]), and an additional eight replicates were subjected to continuous drought, receiving 66% less water than control pots throughout the experiment. Plants were grown to the R8 stage (∼5 weeks) before rhizosphere soil was harvested. Rhizosphere soil was collected in sterile Whirl-Pak bags by uprooting the plants and shaking loose soil from the root system. Any remaining soil adhering to the roots was considered rhizosphere soil. Two rhizosphere soil samples (5 g each) per plant were collected and immediately processed for active and total cell counts (see further details below), while the remaining rhizosphere soil was frozen at −80°C for RNA/DNA extraction. For each plant, DNA was extracted from ∼0.3 g of rhizosphere soil using the DNeasy PowerSoil kit (Qiagen, Carlsbad, CA, USA), while RNA was extracted from ∼2.3 g of rhizosphere soil using the RNeasy PowerSoil kit according to the manufacturer’s instructions.

### Corn and bean soil: microbial cell extraction and active and total cell counts.

For both corn and bean soils, microbial cells were extracted by following a protocol adapted from reference 54 and stained for determination of active and total cell counts. Briefly, soil subsamples (10 g per sample in the corn soil experiment, and two technical replicates of 5 g each in the bean soil experiment) were mixed with 100 ml of chilled sterile phosphate-buffered saline containing 0.5% Tween 20 (PBST). Soil samples were homogenized in a Waring blender (Conair Corporation, East Windsor Township, NJ, USA) three times for 1 min and kept on ice between each blending cycle. Soil slurries were centrifuged at 1,000 × *g* for 15 min, and the supernatant was set aside. The pelleted soil was resuspended in 100 ml PBST, blended for an additional minute, and recentrifuged. The supernatants were pooled and centrifuged at 10,000 × *g* for 30 min. The supernatant was aspirated, and the pellet was resuspended in sterile Milli-Q water (20 ml in the corn soil experiment and 10 ml in the bean soil experiment). Cells were stained for percent activity determination using the *Bac*Light RedoxSensor CTC vitality kit (ThermoFisher Scientific, Waltham, MA, USA). Briefly, 1 ml of cells was stained with 0.38 µl of the DNA stain Syto24 and a 5 mM concentration of the activity stain 5-cyano-2,3-ditolyl tetrazolium chloride (CTC; active community) for 24 h. Stained cells were fixed with 100 µl of 37% formaldehyde, and cell counts were measured on a BD C6 Accuri flow cytometer (Franklin Lakes, NJ, USA), with a cell defined as a fluorescence event greater than 10^3^ on a 490/515-nm filter for Syto24 and a 450/630-nm filter for CTC. Following recommendations by the Michigan State University Flow Cytometry Core, we gated measurements by side scatter values that were <500, which removed particles <1 µm from our measurements.

We calculated the percentage of active cells by dividing CTC counts by Syto24 counts. For each sample in the corn soil experiment, we used a single 10-g sample of soil for microbial cell extraction, which was then split into two technical replicates for staining; results for these two replicates per sample were averaged prior to subsequent analyses to avoid pseudoreplication. For each plant in the bean experiment, we used two replicate 5-g soil samples to give two technical replicate microbial cell extractions. Each of these was then split into three technical replicates for staining. These six replicates per plant were averaged prior to subsequent analyses to avoid pseudoreplication.

### Corn and bean soil: 16S rRNA gene amplicon sequencing.

For both the corn and bean soil experiments, we first verified no DNA contamination in the RNA samples using PCR with 16S primers (55, 56) followed by gel electrophoresis. Next, 3 µl of RNA from each RNA sample was reverse transcribed using the SuperScript RT III kit (Invitrogen) by following the protocol for random hexamers. Nucleic acid concentrations were measured with the Qubit broad-range DNA assay kit (ThermoFisher, Waltham, MA, USA). DNA and cDNA from the bean experiment were diluted to 5 ng μl^−1^ (but were left undiluted in the corn soil experiment) prior to being submitted for sequencing at the Michigan State Genomics Core. cDNA and DNA from both the corn and bean soil experiments were sequenced by the Michigan State University Genomics Core using the dual-index primer pair 515F and 806R (56). Samples were prepared for sequencing by the MSU Genomics Core, which included PCR amplification and library preparation using the Illumina TruSeq Nano DNA library preparation kit. Paired-end, 250-bp reads were generated on an Illumina MiSeq apparatus, and the Genomics Core provided the standard Illumina quality control, adaptor, barcode trimming, and sample demultiplexing.

### Corn and bean soil: bioinformatic and statistical analyses.

The corn and bean soil sequencing data sets were analyzed separately. For each data set, raw reads were merged, quality filtered, dereplicated, and clustered into 97% identity operational taxonomic units (OTUs) using the UPARSE pipeline (57). Taxonomic annotations for OTU representative sequences were assigned in the mothur (58) environment using SILVA rRNA database release 132 (59). OTUs with unassigned taxonomy at the phylum level and OTUs annotated as mitochondria, chloroplasts, or Eukaryota, were removed. All subsequent analyses were performed in R (version 3.5.0 [60]), with ecological statistics performed using phyloseq (version 1.24.0 [61]). Data were visualized using a combination of the R packages ggplot2 (version 2.2.1 [62]), reshape2 (version 1.4.3 [63]), ggpubr (version 0.1.6 [64]), and gridExtra (version 2.3 [65]), and dplyr (version 0.7.5 [66]) was used for data summaries.

First, we examined the prevalence of phantom taxa (i.e., OTUs with detectable RNA reads but no detectable DNA reads [23]) in the corn and bean soil data sets. We calculated the average percentage of OTUs that are phantom taxa in each treatment, as well as the average percentage of OTUs with a single RNA read and no detectable DNA reads in each treatment. We conducted these analyses across a range of subsampling levels (using a step-size of 5,000 reads per sample) to examine the influence of sequencing depth on the prevalence of phantom taxa and used the locally weighted scatterplot smoothing (LOESS) function (67) to generate best-fit lines and confidence intervals. Given the relatively low impact of the subsampling level on the occurrence of phantom taxa, all subsequent analyses were conducted on samples rarefied to the minimum sampling depth in each data set (22,556 reads per sample for corn soil, and 37,815 reads per sample for bean soil).

Given the prevalence and persistence (i.e., their high collective contributions regardless of sampling effort) of phantom taxa in our sequencing data sets, we next compared four different methods for calculating 16S ratios in the presence of phantom taxa. See Fig. 2 for a detailed illustration of the four methods, referred to here as methods 1, 2, 3, and 4 for simplicity. In method 1, each phantom taxon in each sample is set to a 16S ratio of 100 to designate such taxa as active regardless of the threshold 16S ratio activity level chosen, since most studies choose a threshold ratio of less than 10 to designate taxa as active (6, 9, 11). In addition, method 1 sets each taxon in each sample with no detectable reads (those with zero reads in both the RNA and the DNA data sets) to a value of zero, thereby eliminating undefined 16S ratios which arise due to a denominator of zero. In method 2, every instance in which zero DNA reads are detected for a given OTU in a given sample is changed to a value of one to eliminate zeros in the denominator. In method 3, previously used in reference 11, a value of one is added to every OTU in every sample in the DNA data set. This method is meant to eliminate zeros in the denominator (as with method 2) but also to treat every entry in the DNA data set exactly the same. In method 4, previously used in reference 14, a value of one is added to every OTU in every sample in both the RNA and the DNA data sets. As with methods 2 and 3, this method is meant to eliminate zeros in the denominator but also to treat every entry in the entire data set (both RNA and DNA reads) exactly the same. We compared the resulting percentages of activity of the OTUs after using methods 1 to 4 in both the corn and bean soil data sets, using threshold 16S ratios of 1, 2, and 5 for determination of active versus inactive OTUs. Given that methods 1 to 4 captured similar patterns in percent activity across treatments, we conducted all subsequent analyses using the recently published method 3 (11) to calculate 16S ratios.

Next, we examined the relationship between the average number of 16S ribosomal operons per genome for each phylum, obtained from the Ribosomal RNA Database (version 5.4) (42, 68, 69) and the observed 16S ratios in the present study.

Finally, we compared estimates and across-treatment patterns of microbial activity using the 16S ratio (threshold > 1) method to calculate the percentage of active taxa, versus using the cell staining method (CTC counts divided by Syto24 counts) to calculate the percentage of active cells. We also examined both active (CTC) and total (Syto24) counts across treatments to explore the influence that these two values have on the percentage of active cells as calculated by the CTC/Syto24 ratio. Differences among treatments were assessed using analysis of variance (ANOVA), followed by a Tukey *post hoc* test for multiple comparisons. All bioinformatic workflows and custom scripts are available on GitHub (https://github.com/ShadeLab/PAPER_Bowsher_mSystems_2019_16sRatio_CTCstain).

### Data availability.

Corn and bean soil sequencing data were submitted to the NCBI Sequence Read Archive under BioProject accession numbers PRJNA490178 and PRJNA454289, respectively.
