# Supplementary figures and images for: 16S rRNA/rRNA Gene Ratios and Cell Activity Staining Reveal Consistent Patterns of Microbial Activity in Plant-Associated Soil
Source: mSystems. 2019 Apr 2;4(2):e00003-19. doi: 10.1128/mSystems.00003-19 (PMC6445865; doi:10.1128/mSystems.00003-19)

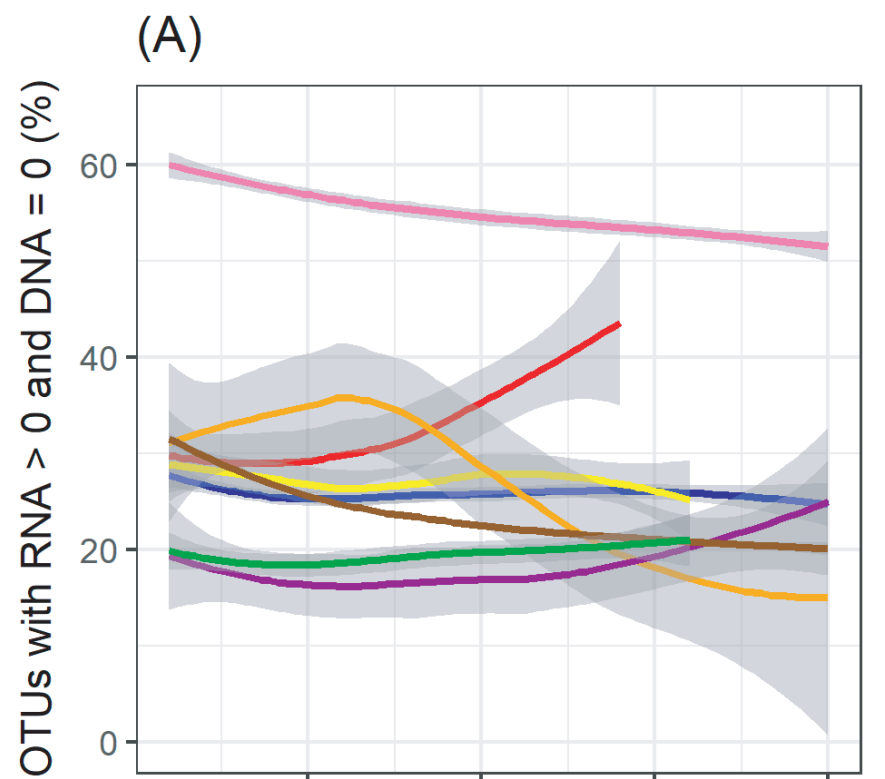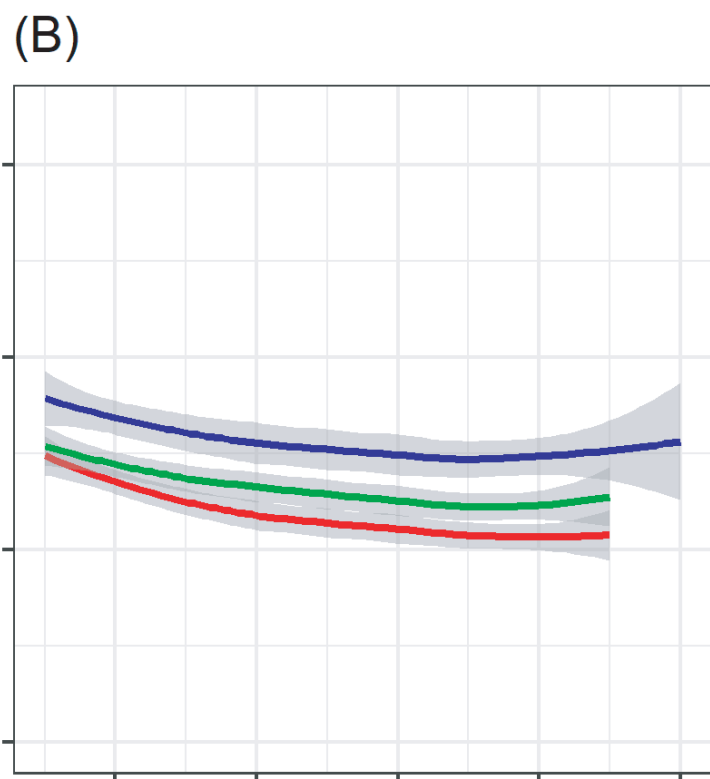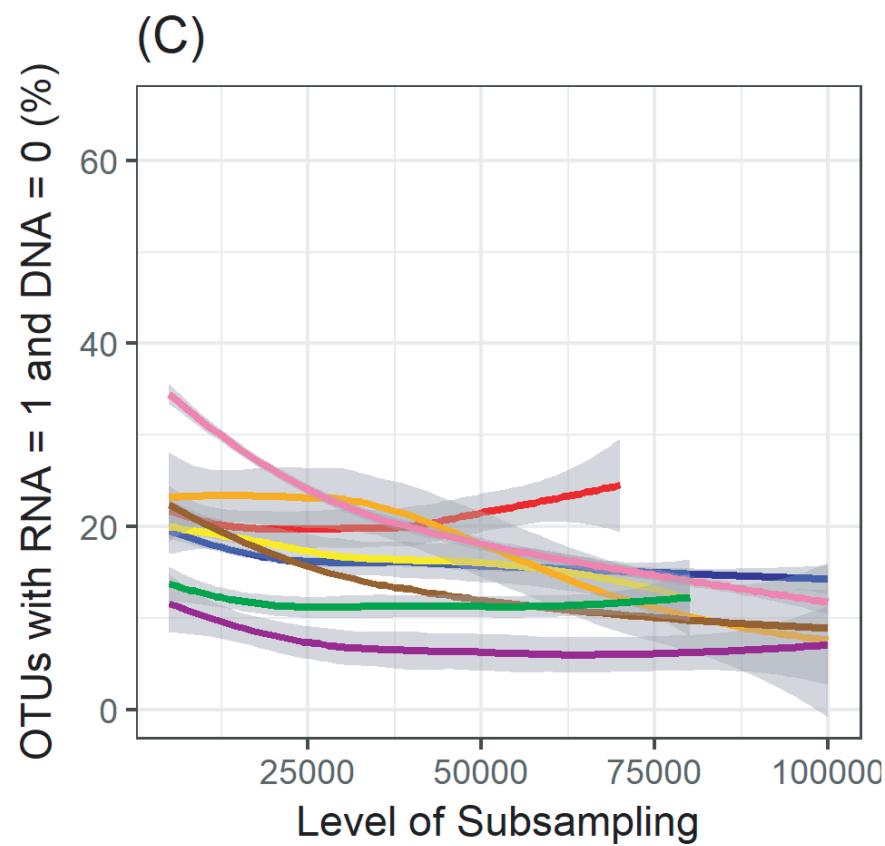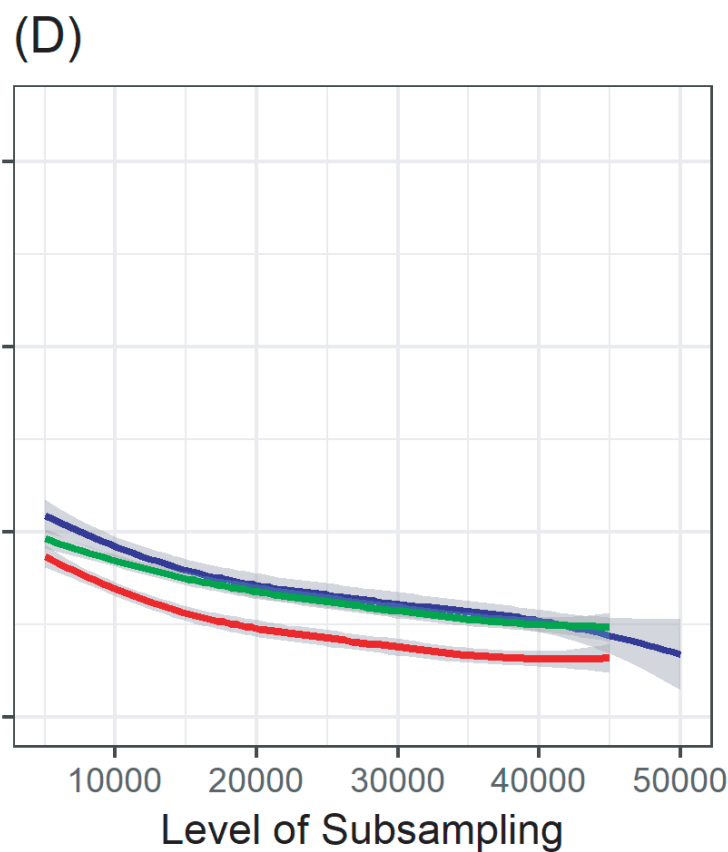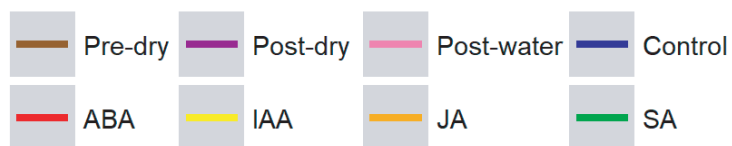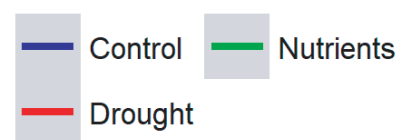

Supplement: FIG S1 [file mSystems.00003-19-sf001.pdf]

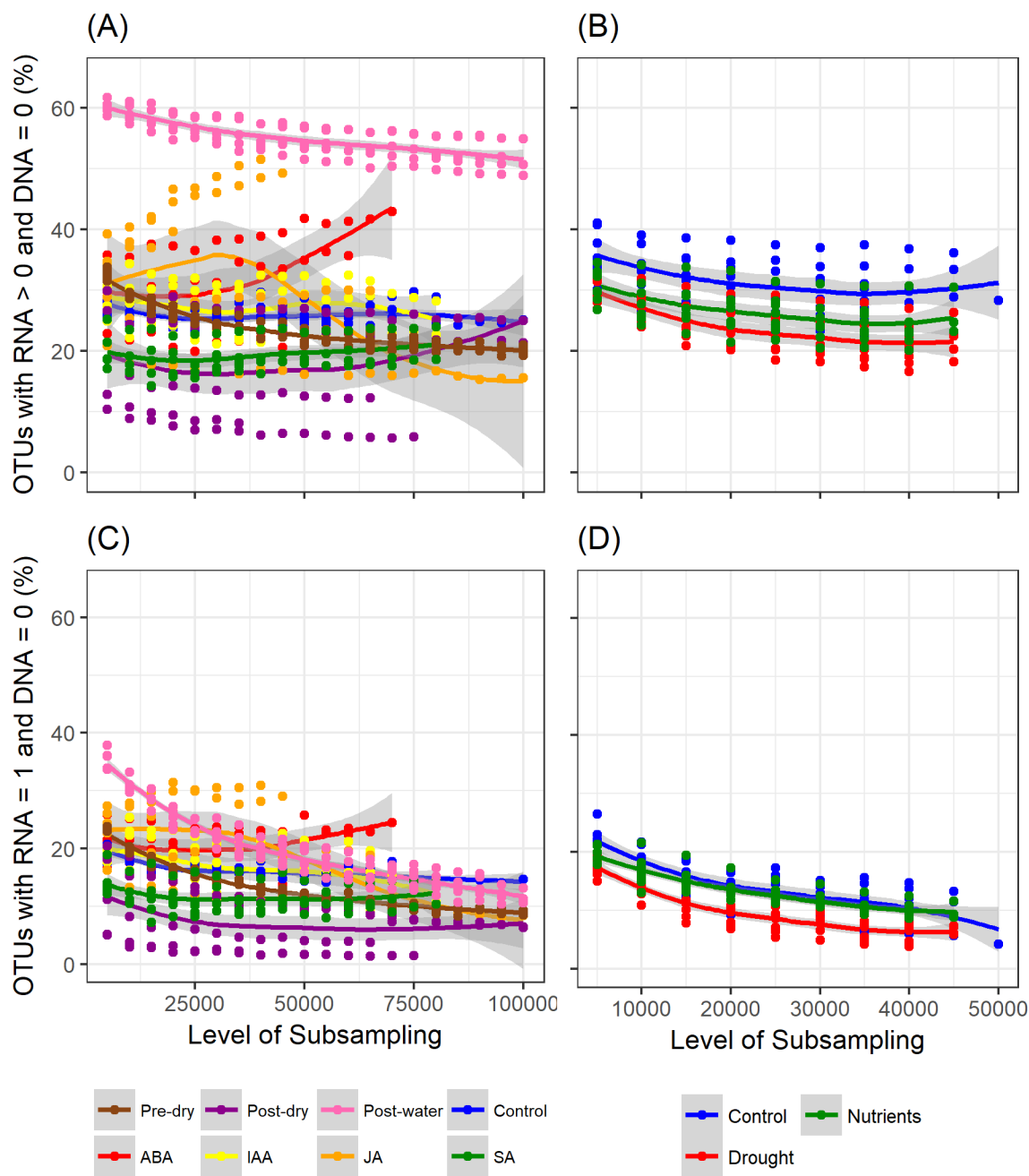

Supplement: FIG S2 [file mSystems.00003-19-sf002.pdf]

(A)

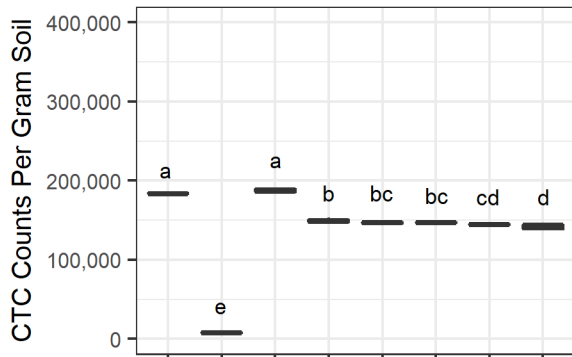

(B)

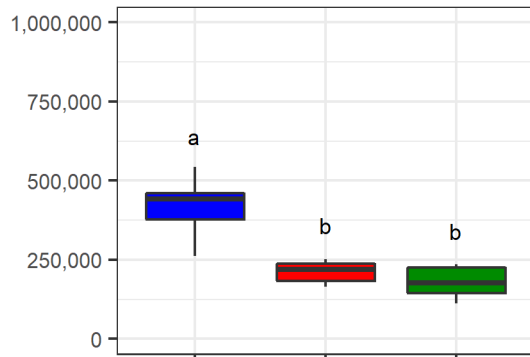

(C)

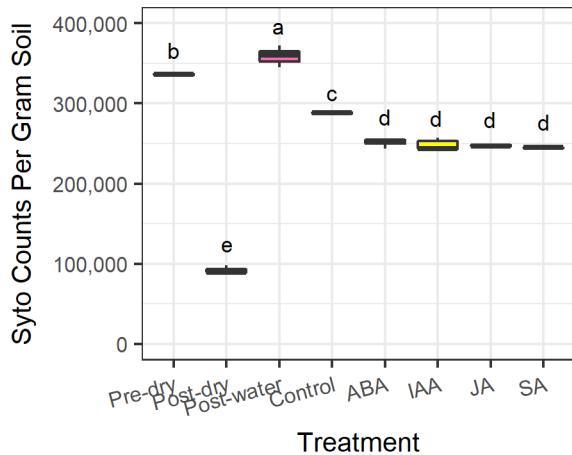

(D)

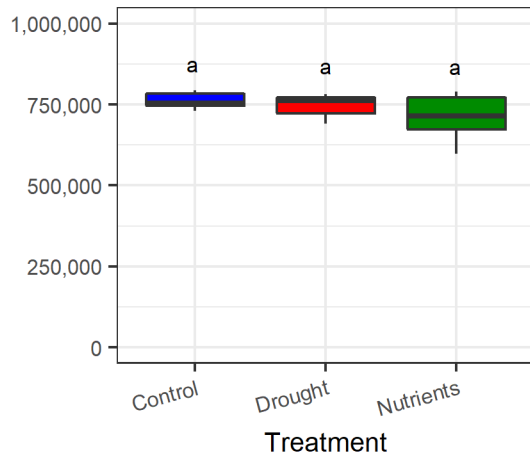

Supplement: FIG S3 [file mSystems.00003-19-sf003.pdf]
